# Association between school-level attributes and weight status of Ghanaian primary school children

**DOI:** 10.1186/s12889-019-6937-4

**Published:** 2019-05-15

**Authors:** Theodosia Adom, André Pascal Kengne, Anniza De Villiers, Thandi Puoane

**Affiliations:** 1 School of Public Health, Faculty of Community and Health Sciences, University of the Western Cape, Cape Town, South Africa; 2 Nutrition Research Centre, Radiological and Medical Sciences Research Institute, Ghana Atomic Energy Commission, Accra, Ghana; 3 Non-communicable Disease Research Unit, South African Medical Research Council, Cape Town, South Africa; 4 Division of Research Capacity Development, South African Medical Research Council, Cape Town, South Africa

**Keywords:** Body mass index, Overweight, Multilevel modelling, School children, Ghana

## Abstract

**Background:**

Little is known about the impact of the school environmental context on the emerging trend of childhood obesity in Africa. We examined the association of the schools’ contextual factors with body mass index (BMI), abdominal obesity and overweight (including obesity) in urban Ghana.

**Method:**

Using cross-sectional data from 543 school children aged 8–11 years attending 14 primary schools, we applied multilevel logistic regressions and linear regression models to investigate the association of child- and school level attributes with overweight, abdominal obesity, and BMI.

**Results:**

We observed significant variance of the random effects of schools in BMI (2.65, *p* <  0.05), abdominal obesity (0.85, p <  0.05), and overweight (1.41, *p* < 0.05), with school contextual levels accounting for 19.7, 20.6, and 30.0% of the total variability observed in BMI, abdominal obesity and overweight respectively. Attending high socioeconomic (SES) level school, private school and school with increased after-school recreational facilities were associated with higher BMI. Children were more likely to be overweight if they attended a high SES level school, had access to healthful foods at school, and after-school recreational facilities. With regards to abdominal obesity, attending a school with increased physical activity facilities decreased the odds of abdominal obesity; however the odds increased if they attended a school with access to after-school recreational facilities.

**Conclusion:**

A number of school-level factors were associated with BMI, overweight and abdominal obesity of children in the present study. Our results provide support for improved school environment to reduce overweight.

## Background

Obesity among children presents a significant public health concern globally [1, 2] making its prevention a priority. It is well established that the aetiology of obesity is multifactorial [3]. Given the limited success of individual behavioural-based interventions to address the increasing prevalence of overweight and obesity [4], it is imperative to consider other factors outside of the individual. The ecological model, which postulates that changes in individual outcomes are influenced not only by individual-level factors such as age, gender, genetics and race/ethnicity but are also largely influenced by the socio-cultural, economic, and environmental context in which the individual lives, has been widely used in health promotion [5–8]. It is useful in providing better understanding of the multiple factors that may either facilitate or serve as barriers to making healthy choices. For the school-going child, the school is considered one important setting for the development of health-related behaviours and outcomes.

Schools are identified as one of the potential settings to deliver nutrition and physical activity interventions aimed at reducing childhood obesity [4, 9] as school children a spend considerable amount of their waking hours in the school settings. Prior research has investigated the associations of school demographics, school physical activity and food policies and resources, school neighbourhood, support for active transport, after-school recreational facilities and programmes in relation with the obesity epidemic [10–15]. Other studies have also examined school environments and health-related behaviours such as nutrition and physical activity [16–19]. The evidence from these studies underscores the importance of the school food and physical activity environments in shaping dietary and physical activity behaviours of school children and adolescents, and subsequently weight status. While the evidence is limited with regards to the contributions of school-level factors to the health-related behaviours and the emerging trend of childhood obesity in Africa, even fewer studies have examined the correlates of obesity in the context of the school environment using a multilevel approach. Moreover, the evidence provided by the aforementioned studies may not be generalisable to low-to-middle income countries like Ghana. Thus it is imperative to investigate the school contextual factors affecting child weight status, to inform the design and implementation of appropriate interventions. The present study aimed to examine the association of the schools’ contextual factors with body mass index (BMI), abdominal obesity and overweight (including obesity) in urban Ghana.

## Methods

### Study design and study population

This was a school-based cross-sectional study with data collected in 543 school children aged 8–11 years in the Adentan Municipality of Greater Accra Region, an urban area in Ghana. We used a two-stage random sampling method to select schools and recruit the study participants. Briefly, 14 schools (eight private and six public) were selected from 148 basic schools (which were either exclusively primary, or primary and Junior High) in the Municipality. Five hundred and sixty-two (562) healthy children aged 8–11 years who provided parental and child approval were enrolled from the selected schools. Anthropometric data was collected by objective methods. Child- and school- level variables were obtained by self-report.

### Outcome variables

The outcome variable was child weight status, defined as BMI, overweight (including obesity), and abdominal obesity.

Weight, height and waist circumference were measured by trained research assistants. Weight was measured twice to the nearest 0.1 kg with a digital scale (Seca 869, GmbH & Co, Germany). Children were weighed in their school uniforms, without shoes while removing extra clothing like jackets, sweaters, and also any heavy objects from the pockets. Height was measured twice to the nearest 0.1 cm with a Shorr Board (Shorr Productions, Olney, MD). Waist circumference was measured with a Seca measuring tape (tension tape) to the nearest 0.1 cm with the participants’ arms relaxed at the sides and following normal expiration. Body mass index (BMI) was computed from the average measurements of weight and height as weight (in kilogramme) divided by height (in metres)^2^. BMI-for-age Z-score (zBMI) was computed with the WHO AnthroPlus v.1.0.4 and used to categorise pupils into overweight (zBMI > = 1.0 SD) or not overweight (zBMI < 1.0 SD) [20]. Waist-to-height ratio (WHtR) was calculated from the average of the height and waist circumference. Abdominal obesity was defined as WHtR ≥0.45 in girls and ≥ 0.46 in boys [21].

### Individual (child) level explanatory variables

Age was computed as the difference of date of birth and date of measurement. Children self- reported their sex by responding to the question “Are you a boy or a girl?” Data on household socioeconomic status (SES) indicators were obtained from self-reports. Household SES was evaluated using variables on source of water and sanitation, and household assets which were subjected to principal component analysis. The first component was extracted to create wealth scores of the household which were then split into three and reported as poor (lowest 40%), middle SES (40%) and rich (highest 20%) households [22]. Child age was modelled as a continuous variable. Two variables for wealth score were included, one continuous and the other categorical.

### School level explanatory variables

The school heads/administrators completed interview-administered questionnaires on perceived built environment of neighbourhood/community surrounding the schools, school food environment and physical activity environment, policies and practices of physical activity and healthy eating. Two variables were used as indicators of school SES; the type of school (private/public) and aggregated wealth scores, computed from household wealth scores of individual children attending the same school. The school type was dummy coded as public = 0 and private = 1. The school-level SES was treated as a continuous variable in the analysis.

To assess the perceived neighbourhood quality, questions were asked on seven selected variables including mixed land use/access, places for walking and bicycling, free or low cost playgrounds or recreational facilities, aesthetic, traffic and crime rate. Four-point Likert scales responses ranging from “strongly disagree [1] to strongly agree [4] were used. Responses were collapsed and dichotomized into strongly disagree/somewhat disagree and strongly agree/somewhat agree. Negatively worded items were reverse coded and a summary score “Perceived neighbourhood quality” was generated from 6 items (Cronbach alpha = 0.81), as one item was constant (that is no variability) such that a higher score indicates favourable environment. Perceived neighbourhood quality was modelled as a continuous variable.

To assess school policies and practices, respondents provided answers to the questions “*Does your school have written policies or practices concerning physical activity?”, “Does your school have written policies or practices concerning healthy eating?”* Options were “*No/not applicable”, “Yes, existing written policies”, “Yes, written policies still under development*” and “*yes, practices.*

The respondents answered the questions: “*Is structured physical activity currently in the weekly timetable for the pupils*”, “*How many sessions per week, and “How long is each physical activity session*”. Minutes spent per week for physical activity was computed from the number of sessions and time spent in physical activity and included as a continuous variable.

Six items were used to assess schools’ support for active transport to and from school. The respondents answered questions on variables on safe walking/bicycling areas, and allowing or encouraging children to use bicycles and protective headgear like helmet. The response options included: no/I do not know (0) and yes [1]. One item “*Identify safe routes to use for walking and cycling to and from school*”, was constant and was therefore excluded from the analysis. A new variable “Support for active transport” was generated from the summary score of the remaining five [5] items (Cronbach alpha = 0.55), where a higher score indicated favourable ratings.

Access to 13 facilities on and off school grounds during school hours were used: fitness room, secure change room lockers, art room and music room, playground equipment like footballs, skipping ropes, basketballs, playgrounds, outdoor sport fields like basketball courts and any paved area for skipping; running tracks, playground equipment like basketballs, footballs, skipping ropes, netballs, gym, dance studio, auditorium for aerobics. A score of 1 was assigned where available, and 0 not available/do not know. Access to facilities on and off school grounds during school hours were compared with not available/do not know. Five [5] items were excluded (fitness room, secure change room lockers, art room and music room because none of the schools had these facilities) and one other item (playground equipment like footballs, skipping ropes, basketballs, etc.) was reported to be available in all 14 schools so was also excluded from analysis. A new quantitative variable “PA facilities index” was generated from the summary score based on seven items (Cronbach alpha = 0.60).

Respondents answered the questions “*Do pupils have access to the cafeterias, school shops, as well as restaurants close to school (within 1 km radius) where they can buy foods or drinks during school hours*?” The available options were yes and no. In addition, a checklist of foods and drinks on sale at the facilities was completed. If an item was available, a score of yes [1], or no (0) was given. Foods were categorised as healthful (raw fruits, raw vegetable salads, cooked meals, 100% fresh fruit juices) and less healthful (chocolates; sweets/toffees; sodas/soft drinks; packaged fruit juices; cakes, cookies, biscuits; chips; sausages rolls, doughnuts and & pies; regular chips & crackers; popcorn; and ice creams) and a composite scores generated for each school with higher scores indicating availability of these foods. The Cronbach alpha coefficient was 0.70 for healthful foods (four items) and 0.76 for less healthful foods (10 items). These were treated as continuous variables in the analysis.

Availability and accessibility of recreational facilities provided by the school, outside school hours was assessed using four items: 1) equipment (e.g., basketballs, skipping ropes, footballs); 2) indoor facilities, 3) outdoor facilities (e.g., playing fields, paved activity areas; and 4) gymnasium. A score of 1 was given where available, otherwise 0. A summary score “After-school recreational facilities” was generated (Cronbach alpha = 0.70) and modelled as a continuous variable.

The Senate Research Committee of the University of the Western Cape and Ethical Review Committee of the Ghana Health Service approved the study. Additional approval was obtained from the Municipal Education Directorate of the Ghana Education Service and from the heads of participating schools while parents or legal guardians of children provided written informed consent. Also, every participating child gave verbal assent after explaining the study.

### Statistical analysis

All analyses were conducted with Stata 13.0. Mean and standard deviation, median and 25-75th percentiles, student t-test and Mann-Whitney test were used for continuous variables while frequencies and percentages, and chi-square (χ^2^) test were used for categorical variables. Mixed effects models were used to account for the hierarchical nature of the data (pupils nested within schools). To estimate the association of binary outcomes, overweight and abdominal obesity, with child- and school level variables, mixed effects logistic regressions models (*melogit* command) with schools as random effects were fitted. The null model (model with no explanatory variables) was first fitted to report the random effects of the schools and also the intraclass correlation coefficient (ICC). Univariable analyses were then performed with each of the explanatory variables, individually. Explanatory variables which tended to contribute to the variability of outcome variables at *p* < 0.20 were selected and included in the multivariable models. The estimated fixed-effects coefficients are reported as odds ratios (ORs), 95% confidence intervals (CI) and standard errors (SE).

For the continuous BMI variable, mixed effects linear regressions models with schools as random effects were performed to estimate the association of BMI with child level and school level explanatory variables. Similar to the mixed effects logistic regressions, the null model was fitted after which three other models, model 2 (individual level variables), model 3 (school level variables) and model 4 (individual and school levels variables) were fitted to estimate the effects of the variables. For model 4, only variables that contributed significantly to the variability in BMI were included. Estimates are maximum likelihood-based using the *mixed* command. Results are reported as estimates with 95% CI and SE. For all analyses, significance was set at *p* < 0.05. Child age and sex were controlled for in all multivariate models.

## Results

Table 1 summarises the descriptive statistics at the individual (child) and school levels. The study reports data from 543 school children (37.6% boys). Half of the children attended private (50.1%) schools with median age 10 (25th -75th percentiles 9, 11) years. The overall mean BMI and WHtR were 17.03 (SD 3.56) kg/m^2^ and 0.43 (SD 0.05). The corresponding prevalence of overweight (including obesity) and abdominal obesity were 16.4 and 18.8%. Children attending private schools had significantly higher BMI and WHtR compared to their peers in public schools (17.72 (SD 4.29) kg/m^2^ vs 16.34 (SD 2.47) kg/m^2^; *p* < 0.0001) and (0.43 (SD 0.06) vs 0.42 (SD 0.03); *p* = 0.026). Additionally, more than two-thirds of the children who were overweight, and 63.7% of those with abdominal obesity were attending private schools.

**Table 1:** Descriptive statistics at the individual and school levels

	Overall	Private	Public	*p*-value
Continuous variables*				
Individual level (*N* = 543)				
Age (years) ^a^	10 (9, 11)	10 (9,11)	10 (9,11)	0.756
BMI (kg/m^2^)	17.03 (3.56)	17.72 (4.29)	16.34 (2.47)	< 0.0001
WHtR	0.43 (0.05)	0.43 (0.06)	0.42 (0.03)	0.026
Household wealth index	0.27 (−1.47, 1.69)	1.10 (−0.11, 2.16)	− 0.87 (−2.43, 0.51)	< 0.0001
School level (*n* = 14)				
School level SES	0.11 (−0.34, 0.79)	0.78 (0.34, 1.27)	−0.34 (−1.73, − 0.20)	< 0.0001
Healthful foods	2.44 (1.06)	3.16 ± 0.85	1.72 ± 0.70	< 0.0001
Less healthful foods	8.24 (2.04)	9.10 ± 1.07	7.37 ± 2.39	< 0.0001
PA facility index	3.54 (1.30)	4.21 ± 1.07	2.87 ± 1.16	< 0.0001
Minutes per week for PA	69.00 (16.43)	76.5 ± 19.31	60.00 ± 0.00	< 0.0001
After-school recreational facilities	0.65 (0.97)	0.93 ± 1.23	0.37 ± 0.48	< 0.0001
Support for active transport	2.59 (1.19)	2.75 ± 1.12	2.43 ± 1.24	0.002
Perceived neighbourhood quality	3.71 (1.83)	3.99 ± 1.11	3.43 ± 2.3	0.0004
Categorical variables*				
Individual level				
Sex				0.833
Boys	37.6 (204)	49.5 (101)	50.5 (103)	
Girls	62.4 (339)	50.5 (171)	49.5 (168)	
Household SES				< 0.0001
Poor	40.1 (218)	26.1 (57)	73.9 (161)	
Middle	40.0 (217)	60.4 (131)	39.6 (86)	
Rich	19.9 (108)	77.8 (84)	22.2 (24)	
Overweight (including obesity)	16.4 (89)	73.0 (65)	27.0 (24)	< 0.0001
Abdominal obesity	18.8 (102)	63.7 (65)	36.3 (37)	0.002
School level				
Physical activity policies and practices	94.3 (512)	47.1 (241)	52.9 (271)	< 0.0001
Nutrition and healthy eating policies and practices	90.4 (491)	44.8 (220)	55.2 (271)	< 0.0001
Physical activity on timetable	81.7 (444)	88.6 (241)	74.9 (203)	< 0.0001
School cafeteria	52.8 (287)	73.9 (212)	26.1 (75)	< 0.0001
School shop	34.4 (187)	100. 0 (187)	–	< 0.0001
Fast food outlets close to school	95.0 (516)	52.7 (272)	47.3 (244)	< 0.0001

*BMI* Body mass index, *WHtR* waist-to-height ratio, *SES* socioeconomic status; * for continuous variables, mean (standard deviation), median (25th–75th percentiles) are reported and percent (frequency) for categorical variables

Nearly all the participating schools (seven private and six public) have some policies and practices on physical activity, which were existing, under development and or undergoing implementation. Policies and practices on nutrition and healthy eating were available in 90.4% (six private and six public) of the schools. Moreover, 81.7% of the children attended schools (seven private and five public) that had structured physical activity in the weekly timetable. Whereas over half (52.8%) of the schools had cafeteria, 34.4% had school shops. All (95.0%) but one private school reported that the communities surrounding the schools have fast food outlets. Overall summary scores for healthful and less healthful foods were 2.44 (SD 1.06) and 8.24 (SD 2.04) respectively. Children attending private schools had more options of both healthful (3.16 vs 1.72 *p* < 0.0001) and less healthful (9.10 vs 7.37; p < 0.0001) food available compared to their counterparts in public schools.

More facilities were available and accessible to children in private schools compared to those attending public schools. The overall mean physical activity facility index, after-school recreational facilities, schools’ support for active transport and perceived neighbourhood quality were: 3.54 (SD 1.30), 0.65 (SD 0.97), 2.59 (SD 1.19) and 3.71 (SD 1.83). Children spent an average of 69.0 min (SD 16.4) weekly on physical activity. Moreover, the PA facility index was higher in private, compared to public schools (4.21 vs 2.87; *p* < 0.0001). On average, children in private schools spent more time per week in physical activities relative to those in public schools (76.5min vs 60.0min; *p* < 0.0001). Also, the summary score of after-school recreational facilities was higher in private compared to public schools (0.93 vs .037; p < 0.0001). Schools’ support for active transport was 2.75 vs 2.43 in private and public schools respectively (*p* = 0.0018). Additionally, the perceived school neighbourhood quality were 3.99 ± 1.11 and 3.43 ± 2.3 (*p* = 0.0004) in private and public schools.

### Factors associated with overweight (including obesity)

Table 2 shows factors associated with overweight. In the null model, the variance of random effects of schools was 1.408 (*p* < 0.05) and the ICC was 0.300, suggesting that 30% (12.8 to 55.4%) of the total variance observed in overweight in the study was at the school level. In univariable analyses, school level SES, school type, availability of cafeteria and shops at the schools, healthful foods and after-school recreational facilities predicted the likelihood of overweight. Children were more likely to be overweight if they attended private school, 4.19 (1.35, 13.00) and a higher level SES school, 2.05 (1.43, 2.94). Availability of school cafeteria and shops significantly increased the likelihood of overweight by 3.82 (1.25, 11.69) and 5.07 (1.74, 14.72) respectively. Similarly, availability of healthful foods increased the odds by 2.31 (1.26, 4.23), as did also after-school recreational facilities 1.85 (1.19, 2.88). In the model mutually adjusted for all significant predictors, and variables that tended to be related to overweight at *p* < 0.2 (less healthful foods and PA facility index) in univariable models, the likelihood of overweight increased by 2.00 (1.15, 3.46) with every unit increase in school-level SES. Moreover, for every extra unit increase in the availability of healthful foods and after-school creational facilities, the odds of overweight increased by 1.58 (1.06, 2.36) and 1.38 (1.01, 1.87) respectively. The school type, school cafeteria and shops were not significant in the adjusted model. Individual level variables were not significantly associated with the odds of being overweight. None of the school variables decreased the likelihood of overweight.

**Table 2:** Individual and school factors associated with overweight

	Univariable models ^a^			Multivariable models ^b^		
	OR (95% CI)	SE	*p*-value	OR 95% CI	SE	*p*-value
Intercept (null model)	0.150 (0.074, 0.303)	0.054				
Age	0.931 (0.710, 1.220)	0.128	0.604	1.031 (0.791, 1.343)	0.139	0.823
Sex (Boys)	0.901 (0.540, 1.502)	0.235	0.689	0.921 (0.551, 1.540)	0.241	0.755
Median household wealth index	1.068 (0.915, 1.246)	0.084	0.404	–	–	–
Household SES						
Poor	1					
Middle	1.398 (0.751, 2.560)	0.442	0.290	–	–	–
Rich	1.220 (0.562, 2.648)	0.482	0.615	–	–	–
School level SES	2.052 (1.433, 2.939)	0.376	**< 0.0001**	1.998 (1.153, 3.462)	0.560	**0.014**
School type (Private)	4.190 (1.350, 13.004)	2.421	**0.013**	0.399 (0.110, 1.442)	0.262	0.161
School cafeteria	3.815 (1.245, 11.690)	2.179	**0.019**	1.329 (0.458, 3.857)	0.722	0.601
School shop	5.067 (1.744, 14.721)	2.757	**0.003**	1.343 (0.557, 3.237)	0.603	0.511
Fast food outlets close to school	1.379 (0.091, 20.851)	1.911	0.871	–	–	–
Healthful foods	2.313 (1.265, 4.229)	0.712	**0.006**	1.577 (1.055, 2.358)	0.323	**0.026**
Less healthful foods	1.274 (0.946, 1.716)	0.193	0.110	0.944 (0.735, 1.214)	0.121	0.656
PA facility index	0.848 (0.672, 1.071)	0.101	0.167	0.835 (0.661, 1.054)	0.099	0.129
Minutes per week for PA	0.980 (0.935, 1.028)	0.024	0.420	–	–	**–**
School support for active transport	0.936 (0.547, 1.603)	0.267	0.810	–	–	**–**
Perceived neighbourhood quality	1.263 (0.845, 1.887)	0.259	0.255	–	–	**–**
After-school recreational facilities	1.849 (1.187, 2.883)	0.419	**0.007**	1.375 (1.013, 1.866)	0.214	**0.041**
Physical activity policies and practices	0.217 (0.020, 2.325)	0.263	0.207	–	–	**–**
Nutrition and healthy eating policies and practices	1.076 (0.131, 8.808)	1.154	0.946	–	–	**–**
Physical activity on timetable	0.571 (0.087, 3.741)	0.547	0.559	–	–	**–**
*Random effects*						
School level	1.408 (0.485, 4.090)	0.766				
ICC	0.300 (0.128, 0.554)	0.114				

^a^ Univariable models using mixed effects logistic regression; ^b^ Explanatory variables with *p*-values less than 0.20 were forced into the same multivariable model (full model). Covariates adjusted for: school level SES, school type, school cafeteria, school shop, healthful foods, less healthful foods, PA facility index, after school recreational facilities, sex and child’s age. *OR* odds ratio, *SE* standard error, *CI* confidence intervals. *PA* physical activity, *SES* socioeconomic status

### Factors associated with abdominal obesity

As shown in Table 3, in the null model, the variance of random effects of schools was 0.85 (*p* < 0.05) and the ICC was 0.206. This indicates that the school level contributes 20.6% (8.3 to 42.4%) of the total variance observed in abdominal obesity in the study sample. Higher school- level SES [1.64 (95% CI: 1.16, 2.32)]; availability of cafeterias [2.66 (1.02, 6.96)] and shops [3.47 (1.41, 8.56)] at the schools; and after-school recreational facilities [1.82 (1.32, 2.49)] were significantly associated with abdominal obesity in the univariable analyses. In the mutually adjusted model, additional variables that tended to be related to abdominal obesity (school type, healthful foods and PA facility index) in the univariable analyses were included. For every extra unit increase in PA facility index, the likelihood of abdominal obesity significantly decreased by 0.78 (0.63, 0.97), whereas the availability and accessibility of after-school recreational facilities increased the odds by 1.64 (1.23, 2.19). Individual level variables were not significantly associated with the odds of abdominal obesity.

**Table 3:** Individual and school factors associated with abdominal obesity

	Univariable model ^a^			Multivariable model ^b^		
	OR (95% CI)	SE	*p*-value	OR (95% CI)	SE	*p*-value
Intercept (null model)	0.209 (0.120, 0.362)	0.059				
Age	1.108 (0.857, 1.432)	0.145	0.433	1.145 (0.891, 1.471)	0.146	0.289
Sex (Boy)	0.879 (0.545, 1.418)	0.214	0.597	0.888 (0.549, 1.437)	0.218	0.629
Median household wealth index	1.018 (0.885, 1.170)	0.072	0.805	–	–	**–**
Household SES						
Poor	1			–	–	–
Middle	1.262 (0.721, 2.211)	0.361	0.416	–	–	–
Rich	1.236 (0.607, 2.517)	0.448	0.559	–	–	–
School level SES	1.636 (1.155, 2.317)	0.290	**0.006**	1.501 (0.951, 2.369)	0.349	0.081
School type (Private)	2.420 (0.880, 6.655)	1.249	0.087	0.489 (0.150, 1.593)	0.295	0.235
School cafeteria	2.661 (1.017, 6.962)	1.306	**0.046**	1.401 (0.678, 2.896)	0. 519	0.362
School shop	3.473 (1.410, 8.555)	1.597	**0.007**	1.234 (0.531, 2.871)	0.532	0.625
Fast food outlets close to school	0.597 (0.075, 4.756)	0.632	0.626	–	–	–
Healthful foods	1.556 (0.968, 2.503)	0.377	0.068	1.253 (0.881, 1.783)	0.225	0.210
Less healthful foods	1.166 (0.910, 1.495)	0.148	0.224			
PA facility index	0.822 (0.663, 1.019)	0.090	0.073	0.782 (0.627, 0.974)	0.088	**0.028**
Minutes per week for PA	0.993 (0.960, 1.027)	0.017	0.694	–	–	**–**
School support for active transport	0.940 (0.614, 1.438)	0.204	0.775	–	–	**–**
Perceived neighbourhood quality	1.128 (0.821, 1.550)	0.183	0.456	–	–	**–**
After-school PA facilities	1.815 (1.323, 2.492)	0.293	**< 0.0001**	1.642 (1.233, 2.187)	0.240	**0.001**
Physical activity policies and practices	0.308 (0.046, 2.081)	0.300	0.227	–	–	**–**
Nutrition and healthy eating policies and practices	0.715 (0.149, 3.427)	0.512	0.675	–	–	**–**
Physical activity on timetable	0.702 (0.157, 3.132)	0.535	0.642	–	–	**–**
*Random effects*						
School level	0.871 (0.299, 2.421)	0.044				
ICC	0.206 (0.083, 0.424)	0.087				

^a^ Univariable models using mixed effects logistic regression; ^b^ Explanatory variables with p-values less than 0.20 were forced into the same multivariable model (full model). Variables adjusted for: school level SES, school type, school cafeteria, school shop, healthful foods, PA facility index, after-school recreational facilities, sex and child’s age. *OR* odds ratio, *SE* standard error, *CI* confidence intervals. *PA* physical activity, *SES* socioeconomic status

### Factors associated with BMI

Table 4 summarises the multilevel analysis of individual and school level variables associated with child BMI. In model 1 (null model), the variance of the random effects of schools was significant (2.65, *p* < 0.05). The ICC was 0.197, indicating that 19.7% of the total variance observed in child BMI existed at the school level. In model 2 (individual level), none of the variables (age, sex, household wealth index) were significantly associated with BMI. At the school level, model 3, school level SES (β = 0.96, *p* < 0.0001), private school (β = 1.74, *p* = 0.028), availability of school cafeteria (β = 1.83, *p* = 0.017) and shops (β = 2.34, *p* = 0.001), healthful foods (β = 0.77, *p* = 0.046), less healthful foods (β =0.38, *p* = 0.048) and after-school recreational facilities (β = 1.134, p < 0.0001) predicted child BMI. In model 4, (individual and school levels), child age (β = 0.40, *p* = 0.008), school level SES (β = 1.02, p < 0.0001), private school (β = − 1.80, *p* = 0.006), and after-school recreational facilities (β = 0.89, p < 0.0001) predicted BMI. At the individual level, none of the child level variables considered in the present study made substantial contributions to the overall variability in BMI. Nonetheless, in model 4, we observed a significant contribution of child age.

**Table 4:** Individual and school factors associated with BMI

	Model 1 Null model		Model 2 Individual		Model 3 School		Model 4 Individual & school	
	Estimate (95% CI)	SE	Estimate (95% CI)	SE	Estimate (95% CI)	SE	Estimate (95% CI)	SE
Fixed effects								
*Individual level*								
Intercept	17.136 (16.233, 18.038)	0.460						
Age			0.304 (−0.002, 0.609)	0.156			**0.396** (0.102, 0.691)*	0.150
Sex (Boys)			0.011 (−0.563, 0.583)	0.293			0.001 (− 0.571, 0.573)	0.292
Household wealth index			0.010 (−0.152, 0.172)	0.083				
Household SES								
Poor (ref)								
Middle			0.113 (−0.547, 0.774)	0.337				
Rich			0.132 (−0.726, 0.990)	0.438				
*School level*								
School level SES					**0.959** (0.481, 1.437)**	0.244	**1.015** (0.499, 1.530)**	0.263
School type (private)					**1.744** (0.185, 3.303)*	0.795	**−1.795** (−3.078, −0.513)*	0.654
School cafeteria					**1.832** (0.324, 3.340)*	0.769	0.742 (−0.330, 1.814)	0.547
School shop					**2.344** (0.926, 3.762)*	0.724	0.276 (−0.781, 1.333)	0.539
Fast foods restaurants close to school					1.101 (−2.401, 4.602)	1.787	–	–
Healthful foods					**0.769** (0.012, 1.526)*	0.386	0.306 (−0.136, 0.748)	0.225
Less healthful foods					**0.382** (0.003, 0.762)*	0.194	−0.094 (− 0.306, 0.119)	0.108
PA facility index					−0.143 (− 0.391, 0.105)	0.126	–	–
Minutes per week for PA					−0.014 (− 0.064, 0.035)	0.025	–	–
School support for active transport					−0.136 (− 0.840, 0.568)	0.359	–	–
Perceived neighbourhood quality					0.176 (−0.327, 0.678)	0.256	–	–
After school recreational facilities					**1.134** (0.562, 1.705)**	0.291	**0.894** (0.525, 1.263)**	0.23
Physical activity policy					−2.957 (−6.088, 0.174)	1.597	**–**	–
Nutrition policy & healthy eating policy					−0.657 (−3.247, 1.933)	1.321	–	–
Physical activity on timetable					−1.098 (3.585, 1.389)	1.269	–	–
*Random effects*								
Individual level	**10.802** (9.574, 12.187)**	0.665						
School level	**2.650** (1.121, 6.265)**	1.163						
ICC	0.197 (0.093, 0.370)	0.070						

Explanatory variables that were significant compared with the empty model were selected for the final model (model 4). Variables included: school level SES, school type, school cafeteria, school shop, healthy food, less healthful foods, after school recreational facilities, sex and child’s age. ^b^ Linear mixed models used, where variables were modelled as fixed effects and school as random effects. *SE* standard error, *CI* confidence intervals. * = *p* < 0.05; ** = *p* < 0.0001

## Discussion

Our results indicate that generally, private schools tended to have facilities that promote healthy choices (both food and physical activity environments), and also unhealthy options (food environment) compared to public schools, which were resource-constrained. The proportions of children with abdominal obesity and overweight (including obesity) were higher in private schools. We found that individual and school level factors are independently and jointly related to BMI, abdominal obesity and overweight in these children. The school context explained between 19.7 and 30.0% of the school level variability in weight status. We found significant associations of child weight status with school type, school-level SES, availability of school cafeterias (providing school meals) and school shops (sale of competitive foods and beverages), healthful foods, less healthful foods, PA facilities index, availability and accessibility of after-school recreational facilities. With respect to individual level variables, only age was significantly related to BMI. School policies and practices on physical activity were unrelated to child weight status.

School type explained the highest percentage of variability in individual child BMI. We observed that children attending private and higher SES schools have higher BMI, and increased odds of abdominal obesity and overweight compared to their peers attending public and lower SES schools. When controlling for individual and school-level variables, the association with school-level SES was seen with BMI and overweight, but not abdominal obesity. The school type continued to be linked with only BMI, but the direction of the association changed such that children attending private schools had lower BMI compared to their counterparts attending public schools. Our findings paralleled those from prior research among African populations that suggested that attending affluent, private or high SES school increases the odds of overweight and obesity [23, 24]. On the other hand, studies conducted in the US [14, 25] found that children attending higher SES schools had lower BMI. Higher SES schools tend to have more resources that would promote healthy behaviours and hence lower the odds of overweight and obesity in developed countries.

School food environment has a significant impact on children’s eating behaviours [16] and subsequently body weight [10, 12], as more than one-third of the daily caloric intake occurs while at school [26]. Our results suggest that over one-third of the children have access to school cafeterias and shops with a wide selection of both healthful and less healthful foods options, although there were fewer healthful options. This suggests the nutrition and healthy eating policies and practices were poorly implemented or enforced. We observed that the availability of less healthful foods was positively associated with BMI but not overweight and abdominal obesity; and the association was no longer significant after controlling for school-level SES, school type, cafeterias, shops, healthful foods, after-school recreational facilities, age and sex.

This analysis adds to an earlier study that found that less healthful foods at school was positively associated with higher BMI and obesity/overweight [10, 12]. Prior research indicated that the absence of school shops and snack bars and also limiting the availability of less healthful foods in school shops were associated with reduced intake of sugar-sweetened beverages and energy dense snacks [16, 26]. We observed that the availability of healthful foods tend to increase the risk of overweight, abdominal obesity and high BMI, which is counterintuitive. We had expected the availability of healthful foods to be associated with healthy dietary intake, and with lower risk of obesity. This finding indicates that healthful foods did not protect the children from poor dietary habits. Given the increased exposure to less healthful options, food preference, the main determinant of food intake in children [27], could have contributed to poor dietary behaviours, thus the overall intake of these foods would displace the healthy options in the diets. Notably, the home food environment, an equally important context for developing food preferences and dietary habits in children [28–30], was not captured in the present analyses.

Among the physical activity environments considered in the present study, only PA facility index was significantly associated with abdominal obesity, but not overweight or BMI. Availability and accessibility to play equipment and resources provide children with the opportunities to be physically active during school hours. Children attending schools that had more PA facilities both on and off grounds tend to have lower odds of abdominal obesity than their counterparts who were attending schools that were poorly-resourced, consistent with existing research [11]. In the aforementioned study conducted among adolescents, students who had access to these facilities were less likely to be overweight. Some existing research has linked the provision of recreational facilities both at the school level and school community to increased physical activity [17] while results from other studies were mixed [13]. Evidence from a meta-analysis by Morton and colleagues [13] indicated that the association was with activity-specific facilities but not the overall physical activity resources. Nonetheless, the evidence linking school physical activity facilities to child weight status has been inconsistent. Researchers found little or no significant association between physical activity resources and programmes and weight status in adolescents attending middle to high schools [31].

After-school programmes may promote increased participation in extracurricular physical activity and related-health outcomes among children and adolescents [19]. Our results suggest that children attending schools with increased access to after-school recreational facilities had higher risk for increased BMI, overweight and abdominal obesity. The counterintuitive finding could be due to some individual and school level confounding variables that were not considered in the analysis. We did not report on after-school programmes being offered to the children. One possible explanation is that the availability of these facilities were not associated with organised sports with trained physical education staff to supervise the children. Moreover, the need for academic excellence may lead to more time being allocated to extra classes than extra sporting activities. Thus children may not have adequate time to engage in after-school programmes despite the facilities being available.

Strengths of the present study include the application of multilevel modelling to account for the hierarchical nature of the data; and use of objective measures of height and weight. There are limitations of the present work which should be noted. The cross-sectional design of the study precludes the inferences of causal relationships. At best, we could say that associations exist between the examined variables and the outcome variables. Another potential source of limitation was that maternal educational, a significant predictor of child weight was not controlled for in the analysis. We did not report on dietary and physical activity behaviours of the children at school which could provide explanations to the observed variability since the present work focused on school environment and weight outcomes.

## Conclusion

A number of school-level factors were associated with BMI, overweight and abdominal obesity of the children in the present study. Unhealthy weight status was significantly higher in children in private compared to public schools. The fact that children spend significant amount of time in schools could present a window of opportunity to impact healthy lifestyle behaviours which are likely to be maintained through adulthood thereby reducing the prevalence of overweight. Our results add to the limited and inconsistent findings in this area and provide support for improved school environment to reduce the overweight epidemic.

## Abbreviations

BMI: Body mass index
CI: Confidence intervals
IAEA: The International Atomic Energy Agency
ICC: Intraclass coefficient
OR: Odds ratio
PA: Physical activity
SE: Standard error
SES: Socioeconomic status
WHO: World Health Organisation
WHtR: Waist-to-height ratio
zBMI: BMI-for-age
β: Beta coefficient
